# In Vitro Pharmacological Activities and GC-MS Analysis of Different Solvent Extracts of* Lantana camara* Leaves Collected from Tropical Region of Malaysia

**DOI:** 10.1155/2015/506413

**Published:** 2015-12-09

**Authors:** Mallappa Kumara Swamy, Uma Rani Sinniah, Mohd. Sayeed Akhtar

**Affiliations:** ^1^Department of Crop Science, Faculty of Agriculture, Universiti Putra Malaysia (UPM), 43400 Serdang, Selangor, Malaysia; ^2^Institute of Tropical Agriculture, Universiti Putra Malaysia (UPM), 43400 Serdang, Selangor, Malaysia

## Abstract

We investigated the effect of different solvents (ethyl acetate, methanol, acetone, and chloroform) on the extraction of phytoconstituents from* Lantana camara* leaves and their antioxidant and antibacterial activities. Further, GC-MS analysis was carried out to identify the bioactive chemical constituents occurring in the active extract. The results revealed the presence of various phytocompounds in the extracts. The methanol solvent recovered higher extractable compounds (14.4% of yield) and contained the highest phenolic (92.8 mg GAE/g) and flavonoid (26.5 mg RE/g) content. DPPH radical scavenging assay showed the IC_50_ value of 165, 200, 245, and 440 *μ*g/mL for methanol, ethyl acetate, acetone, and chloroform extracts, respectively. The hydroxyl scavenging activity test showed the IC_50_ value of 110, 240, 300, and 510 *μ*g/mL for methanol, ethyl acetate, acetone, and chloroform extracts, respectively. Gram negative bacterial pathogens (*E. coli* and* K. pneumoniae*) were more susceptible to all extracts compared to Gram positive bacteria (*M. luteus*,* B. subtilis*, and* S. aureus*). Methanol extract had the highest inhibition activity against all the tested microbes. Moreover, methanolic extract of* L. camara* contained 32 bioactive components as revealed by GC-MS study. The identified major compounds included hexadecanoic acid (5.197%), phytol (4.528%), caryophyllene oxide (4.605%), and 9,12,15-octadecatrienoic acid, methyl ester, (Z,Z,Z)- (3.751%).

## 1. Introduction

Nature has existed as a source of almost all drugs for many years and natural products were the only source of medicine for mankind ever since the ancient period. Herb based products play an important role in primary human health care as the majority (80%) of the global population rely on traditional medical practices [1, 2]. Most of the modern drugs are derived either from plant sources or from their derivatives for various medicaments and are extensively used in the pharma industry [2]. In addition to the prevailing health problems, emerging infectious diseases and disorders have seriously caused the world population to suffer with a high mortality rate. It is reported that about 50% of all fatality occurring in tropic countries is mainly due to the current infectious diseases [3]. Also, increase of antimicrobial resistance among the pathogens is a rising problem which is challenging the scientific advancement of the medical world [4]. This situation has prompted researchers to develop efficient new antimicrobial agents. Therefore, exploration of natural products as leads to discover new drug molecules is continuously made to understand their therapeutic potential with special reference to biological activities, efficiency, and safety aspects. Exploration of medicinal plants for curative purposes is mainly based on the available traditional information from the experts and local population [5, 6].

*Lantana camara* L. is a medicinal aromatic plant that belongs to the family Verbenaceae and occurs in most parts of the world as an evergreen notorious weed species. It is also considered as an ornamental garden plant. It is widely used in different traditional medical practices for treating various health problems. Different parts of the plant are used in treating various human ailments such as measles, chicken pox, tetanus, malaria, cancers, asthma, ulcers, fevers, eczema, skin rashes, cardiac disorders, and rheumatism [7, 8]. Also leaf extracts and essential oil of* L. camara* leaves possess larvicidal activities, antioxidant, anti-inflammatory, analgesic, antidiabetic, hypolipidemic, anthelmintic, wound healing, and antipyretic properties [9, 10]. The therapeutic potential of the plant is due to the occurrence of many bioactive phytocompounds such as terpenoids, alkaloids, flavonoids, phenolics, glycosides, and steroids as major phytoconstituents [11]. Some of the important bioactive compounds include quercetin, isorhamnetin, oleanolic acid, lantadene A, *β*-sitosterol pomonic acid, camaric acid, verbacosides, lantanoside, linaroside, octadecanoic acid, palmitic acid, and docosanoic acid. The essential oil from the leaves is rich in monoterpenes and sesquiterpenes [11–13]. Various factors including genetic, geographical location, plant parts, and environmental factors have been shown to influence the accumulation of phytochemical contents in different parts of* L. camara* and its essential oil composition [14–16]. Also, occurrence of varietal differences in phytoconstituents of* L. camara* has been documented by Sharma et al. [17]. More recently, in vitro study indicated the existence of chemical differences in methanol leaf extract of four varieties of* L. camara* collected from India and their antioxidant property was found to differ [8]. Certainly, more research efforts should be carried out to explore the potential benefits of* L. camara* for treating various health problems. Therefore, the present investigation was aimed at determining the phytochemical constituents and antioxidant and antimicrobial activities of different solvent extracts of* L. camara* leaves collected from the tropical region of Malaysia. Moreover, the bioactive components of the extracts were also identified using GC-MS analysis.

## 2. Materials and Methods

### 2.1. Plant Collection

*L. camara* plant material was collected from the forest area near Universiti Putra Malaysia, Serdang, Selangor, Malaysia, during the month of May 2015. Plant material was authenticated by N. A. P. Abdullah, Department of Crop Science, Universiti Putra Malaysia, Malaysia, and the voucher specimen (LC-102015) was deposited at the department. The leaves were detached from the collected materials, washed with water and dried under shade for 1 week, and finely powdered using electric blender. The powdered material was kept at room temperature for future use.

### 2.2. Preparation of Extracts

Five grams of powdered leaves was kept in a beaker to which 100 mL of various organic solvents (ethyl acetate, methanol, acetone, and chloroform) was added and thoroughly shaken. Later the mixture was placed at room temperature for 48 hrs and stirred 2-3 times a day. After filtering the mixture, the filtrate was evaporated to dryness using Rotavapor. The final extracts were weighed to determine the yield (%) and the dried extracts were stored at 4°C in a refrigerator for further studies.

### 2.3. Phytochemical Screening

The presence of various phytochemical constituents such as alkaloids, saponins, flavonoids, phenolics, anthraquinones, tannins, cardiac glycosides, steroids, and terpenoids was screened qualitatively by using standard procedures [18–20].

### 2.4. Determination of Total Phenolic and Flavonoid Contents

Total phenolic contents present in different solvent extracts were determined by using FC (Folin-Ciocalteu) colorimetric method as detailed by Salar and Seasotiya [21]. About 0.1 g of dried extract was suspended in 1 mL of the respective solvents and 0.1 mL of this solution was mixed thoroughly with 1 mL of sodium carbonate (20%) solution and 0.5 mL of 50% FC reagent. Thereafter, the solution was allowed to remain at room temperature for about 20 min to observe the color change. The absorbance was taken against the blank (water) at 730 nm. Using different concentrations of gallic acid, a standard calibration curve was generated. Total phenolic content was represented as mg of gallic acid equivalents (GAE) per gram of dried extract.

Total flavonoids content present in different solvent extracts was verified by the modified procedure of Zhishen et al. [22] using colorimeter. Briefly, 2 mL of distilled water was mixed well with 0.5 mL of solvent extract. Thereafter, 150 *μ*L of NaNO_2_ solution (5%) was added and kept for 5 min at room temperature. It was followed by adding 600 *μ*L of AlCl_3_ (10%) and 2 mL of NaOH (4%). After mixing thoroughly, the solution was made up to 5 mL with distilled water and set aside for 15 min at room temperature. By using water as blank, absorbance was measured at 510 nm. A standard calibration curve was generated using different concentrations of rutin. Total flavonoid content was articulated as mg of rutin equivalent (RE) per gram of dried extract.

### 2.5. Antioxidant Activity

#### 2.5.1. Free Radical Scavenging Activity

The antioxidant activity of each extract was assessed using DPPH (1,1-diphenyl-2-picrylhydrazyl) free radical scavenging assay as explained by Mohanty et al. [23] with little modifications. In short, 0.3 mL of different extract at varied concentrations (100–1000 *μ*g/mL) was mixed with 2 mL of 0.1 mM DPPH solution and incubated for 30 min under dark conditions at room temperature. Afterwards, using UV-visible spectrophotometer, the absorbance was taken at 517 nm against methanol (blank) while ascorbic acid served as a standard sample. The reaction mixture showing lower absorbance is an indication of higher activity of radical scavenging. The inhibition percentage of free radical scavenging was calculated based on the following formula:

(1) DPPH scavenging activity  %=Acontrol−AsampleAcontrol×100.

For each extract, IC_50_ values (the minimum quantity of extract necessary for scavenging free radicals up to 50%) were calculated from the standard plot.

#### 2.5.2. Hydrogen Peroxide (H_2_O_2_) Scavenging Activity

The potential capacity of different solvent extracts to scavenge H_2_O_2_ was carried out by the method outlined by Mohanty et al. [23]. In brief, various quantities of plant extract (100–1000 *μ*g/mL) were prepared as detailed in previous experiment. By using phosphate buffer (pH 7.4), H_2_O_2_ solution (4 mM/L) was prepared. Varied amounts of plant extracts were mixed with 0.6 mL of H_2_O_2_ solution and kept for 30 min incubation at room temperature. The activity of H_2_O_2_ was analyzed by taking absorbance at 230 nm using phosphate buffer without H_2_O_2_ as blank solution. H_2_O_2_ scavenging activity (%) was calculated by using the following equation:

(2) H2O2  scavenging activity  %=Acontrol−AsampleAcontrol×100.

For each extract, IC_50_ value (the minimum quantity of extract necessary for scavenging free radicals up to 50%) was calculated from the standard plot.

#### 2.5.3. Determination of Antibacterial Activity

The antibacterial activities for the extracts were evaluated by using disc diffusion method. The experiment included both Gram positive (*Micrococcus luteus*,* Bacillus subtilis*, and* Staphylococcus aureus*) and Gram negative (*Escherichia coli* and* Klebsiella pneumoniae*) pathogenic strains of bacteria. One mg of each extract was dissolved in 1 mL of DMSO (dimethyl sulfoxide) and about 2.5, 5.0, and 10 *μ*L of this solution were impregnated on sterilized filter paper discs (6 mm size). The discs were kept on the nutrient agar medium preinoculated uniformly with the known bacterial culture. The discs soaked with chloramphenicol (50 *μ*L of 50 *μ*g/mL) served as positive control while discs soaked with 50 *μ*L DMSO served as negative control. All culture plates were kept in an incubator at 37°C for 24 h and the bacterial inhibition zone was recorded and expressed in mm. For each bacterial strain, the test was repeated 3 times.

### 2.6. GC-MS Analysis

GC-MS analysis of the active methanol extract of* L. camara* was carried out by using the GC-MS instrument (Model GCMS-QP2010 Ultra, Shimadzu Co., Japan) equipped with a capillary column DB-1 (0.25 *μ*m film × 0.25 mm i.d. × 30 m length). The instrument was operated in electron impact mode at ionization voltage (70 eV), injector temperature (230°C), and detector temperature (280°C). The carrier gas used was helium (99.9% purity) at a flow rate of 1 mL/min and about 1 *μ*L of the sample was injected. The oven temperature was initially programmed at 80°C (isothermal for 5 min.) and then increased to 200°C at 5°C/min and finally to 280°C at 5°C/min (isothermal for 16 min). The identification of compounds from the spectral data was based on the available mass spectral records (NIST and WILEY libraries).

### 2.7. Statistical Analysis

All the data measured in each experiment included 3 replications (*n* = 3) and the results were represented as mean ± SD. The one-way analysis of variance (ANOVA) was performed to compare the data and Tukey's test was used to find out the statistically significant differences at *p* < 0.05 using statistical software, GraphPad Prism version 5.0.

## 3. Results and Discussion

The results of preliminary investigation on the phytochemicals present in different solvent extracts of* L. camara* are presented in Table 1. Different phytocompounds such as alkaloids, saponins, flavonoids, phenolics, anthraquinones, tannins, cardiac glycosides, steroids, and terpenoids were detected in the crude extracts. Flavonoids, phenolics, and cardiac glycosides were noticed in all solvent extracts used. Methanol extracts with 7 phytoconstituents were the best among the organic solvents evaluated in our study. Acetone extract showed the presence of 6 phytoconstituents while ethyl acetate and chloroform extracts contained 5 phytocompounds. All these identified phytochemicals are known to have a wide range of biological activities including antibacterial, antifungal, antiviral, antioxidant, and cytotoxic properties [19]. Understanding the occurrence of phytochemicals in medicinal plants is advantageous and presently, the discovery of new drug compounds or lead molecules from plants is mainly based on the systematic examination of different plant extracts or plant based products. Also, this preliminary knowledge can decipher a new source for economically valued chemical compounds [1, 23].

The weight of the leaf extracts and their yield obtained from different solvent extracts of* L. camara* are presented in Table 2. Different solvents showed a significant influence on the total dry weight and yield of the extracts. Relatively, the extract from methanol resulted in superior extraction yield (14.4%) with 721.3 ± 1.5 *μ*g of dry weight. The recovery of extractable constituents from different extracts remained in the following order of methanol > ethyl acetate > acetone > chloroform. Our results are in conformity with previous studies supporting the use of methanol as the best solvent to recover higher extractable compounds from various medicinal plants [23, 24]. Similarly, Anwar et al. [16] have stated that methanol as the best solvent for extraction from* L. camara*. Also, methanol was commonly employed by other researchers in* L. camara* for various biological studies [8, 25–27]. However, literature study shows that there are no available reports on the comparative yield analysis obtained from different solvent extracts till date. The existence of significant differences of dry weight of the extracts and yields between various organic solvents can be because of different polar nature of the solvents tested [24].

Polyphenols and flavonoids are the plant secondary metabolites occurring in several medicinal plants known to possess antimicrobial, antioxidant, antispasmodic, antidepressant, antitumor, antimutagenic, anti-inflammatory, and many other biological activities [27, 28]. In plants, these phenolic compounds provide defense against various pathogens, regulate cell division and growth, and help in pigmentation and many other metabolic pathways [29]. Therefore, we investigated the occurrence of total phenolic and flavonoid content in different organic solvent extracts (Table 3). The results clearly indicated the existence of statistically significant differences (*p* < 0.05) among the different extracts. The highest phenolic content (92.8 ± 1.7 mg GAE/g) and flavonoid content (26.5 ± 0.5 mg RE/g) were observed in the methanol leaf extract of* L. camara*. On the other hand, ethyl acetate, acetone, and chloroform extract contained phenolic content of 75.6 ± 0.9, 62.9 ± 1.7, and 33.7 ± 0.5 mg GAE/g, respectively. Total flavonoid content in ethyl acetate, acetone, and chloroform extract was found to be 16.7 ± 0.6, 20.6 ± 0.3, and 21.2 ± 0.8 mg RE/g, respectively. Similarly, previous studies have shown the occurrence of rosmarinic and caffeic acid as the major phenolic compounds and few flavonoids such as 3,7-dimethoxy-, 3-methoxy-, and 3,7,4′-trimethoxyquercetin, hispidulin, 3, pectolinarigenin 7-O-*β*-D-glucoside, and camaraside glycoside were evident in the plant extract of* L. camara* [7, 11, 26]. Due to high polarity, methanol was found to exhibit better efficiency in extracting various polar phytocompounds (phenolics and flavonoids) from the leaves of* L. camara*. Both total phenolic and flavonoid contents obtained in our study were relatively much higher than the quantity obtained by earlier researchers in the same species but collected from geographically distant places [8, 16, 26]. However, total phenolic content was much lesser than the quantity (245.5 ± 3.5 mg gallic acid/g) as reported by Mahdi-Pour et al. [15] from methanol leaf extract of* L. camara* located in Kedah, Malaysia, and this difference can be attributed the influence of environmental factors and geographical location.

**Figure 1:**
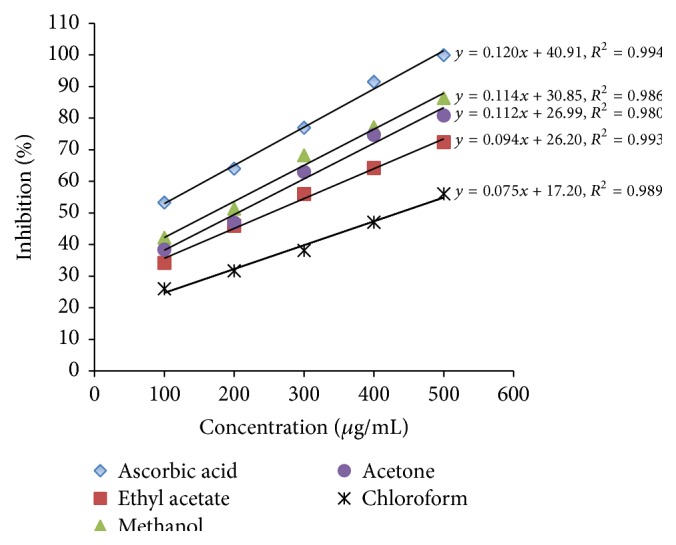
DPPH scavenging activities of various solvent extracts of* L. camara*.

The formation of increased free radicals in human body may cause cell damage andinduces various disorders such as cancer, myocardial infarction, atherosclerosis, and neurodegenerative disorders. However, antioxidant compounds derived from natural source or plants can repair these free radicals formed in cells and thereby, antioxidants are very useful in preventing various disorders [2, 15, 23]. The antioxidant capacities of either natural products or crude plant extracts are usually evaluated by making use of DPPH radical scavenging test [23]. In principal, the test depends on the capacity of DPPH free radicals reacting with plant metabolites such as phenolic and flavonoid compounds (H^+^ donors) occurring in the sample. After the reaction, DPPH solution turns from purple to yellow color due to acquiring of a proton from the donor species. The intensity of color change directly relates to the scavenging ability of the biological sample [27].  Figure 1 shows the DPPH scavenging activity of* L. camara* leaf extracts in comparison to positive control (ascorbic acid). In all the solvent extracts, radical scavenging activity was found to be concentration dependent. The highest percentage of scavenging activity (86.4 ± 0.2 *μ*g/mL) was observed in methanolic leaf extracts at 500 *μ*g/mL concentration. The next best solvent extract was found to be acetone (80.5 ± 0.3 *μ*g/mL) followed by ethyl acetate (72.4 ± 0.3 *μ*g/mL) and chloroform (526.1 ± 0.3 *μ*g/mL). However, the activities of all extracts were inferior to that of the ascorbic acid standard. The IC_50_ values of DPPH free radical scavenging activity were found to be in the following order: ascorbic acid (80 *μ*g/mL) > methanol extract (165 *μ*g/mL) > ethyl acetate (200 *μ*g/mL) > acetone (245 *μ*g/mL) > chloroform (440 *μ*g/mL). These results are on a par with or even superior to that of reports by other researchers on several other crude extracts of the plant evaluated under the same conditions using DPPH assay [27]. The higher antioxidant potential of plant extracts is correlated to the occurrence of many antioxidant compounds especially polyphenols [23]. Although the antioxidant activity of* L. camara* leaf extracts by using DPPH assay was reported by earlier researchers [15, 16, 26], none of them compared the effects of different solvents on antioxidant potential. In our study, due to higher solubility of antioxidant compounds, the methanol extract exhibited increased radical scavenging activity compared to other solvent extracts. Similarly, other researchers have stated that free radical scavenging potential of plant extracts depends mainly on the occurrence of bioactive compounds, particularly polyphenols [8, 30].

**Figure 2:**
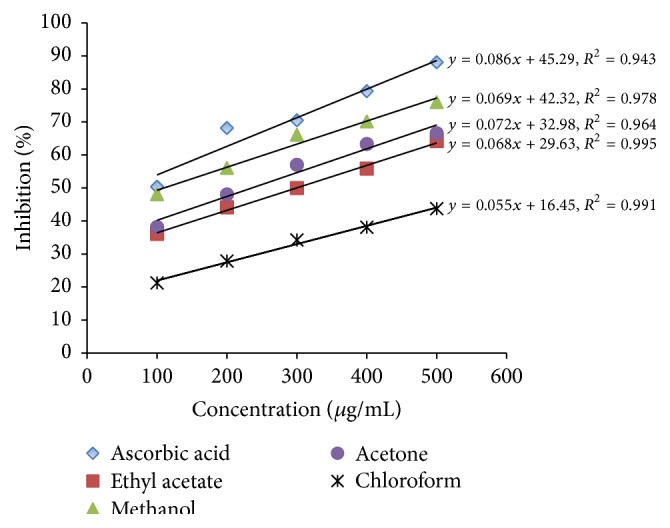
H_2_O_2_ scavenging activities of various solvent extracts of* L. camara*.

In biological system, a large quantity of hydroxyl radicals is formed due to activation of immune cells which are known to be highly toxic radicals and causes extreme damage to all molecules occurring in live cells. These radicals can trigger cell toxicity and mutagenesis by damaging DNA nucleotides [8, 31]. Therefore, measuring hydroxyl radical scavenging activity can provide good information on the antioxidant potential of* L. camara* leaf extracts obtained by different solvents. The results of hydroxyl radicals inhibition obtained from our study are depicted in Figure 2. It is evident that, irrespective of the organic solvents used for extraction, the percent inhibition of hydroxyl radicals increased with the concentration of the extracts. At higher concentration of 500 *μ*g/mL, the percentage of hydroxyl scavenging activity for ascorbic acid, ethyl acetate, methanol, acetone, and chloroform extracts was found to be 88.1 ± 0.4, 64.2 ± 1.0, 76 ± 0.3, 66.6 ± 0.5, and 43.7 ± 0.2%, respectively. The IC_50_ value was found to be in the following order of ascorbic acid (60 *μ*g/mL) > methanol (110 *μ*g/mL) > ethyl acetate (240 *μ*g/mL) > acetone (300 *μ*g/mL) > chloroform (510 *μ*g/mL). The lower the IC_50_ value, the higher the scavenging activity and hence, methanol extract was found to possess superior antioxidant potential compared to other extracts tested. However, their effect was considerably lesser than the standard, ascorbic acid. These results completely corroborate the earlier reports that have proved the existence of correlation between the antioxidant property and the concentration and composition of different plant metabolites occurring in the extracts [23, 32]. In our study, methanol extracts contained significantly higher quantity of phenolics (92.8 ± 1.7 mg GAE/g) and flavonoids (26.5 ± 0.5 mg RE/g) and hence possessed superior antioxidant potential compared to other solvent extracts.

At present, the increased prevalence of deadly diseases and microbes adapting to antibiotic resistance is a great concern in the medical world [33]. Hence, more research interest is shown by medical community towards the development or discovery of novel antimicrobial agents. Due to the severe side effects of several synthetic antibiotics, research preference is mainly focused on discovering plant based natural drugs [5, 34]. Since* L. camara* possessed numerous secondary metabolites, we evaluated the effects of their different solvent extracts against some common human pathogenic bacterial strains. The results of our study revealed that all solvent extracts of* L. camara* were effective against both Gram positive and Gram negative bacterial strains tested, but their efficacy varied (Table 4). With the increase in the concentration, there was an enhanced antibacterial activity irrespective of the solvent used for extraction. Gram negative bacteria were more susceptible to all extracts compared to Gram positive bacteria. In general, Gram negative bacteria are known to exhibit high resistance towards wide range of chemical agents and antibiotics compared to Gram positive bacteria. Besides, Gram negative bacteria are reported to be the most prevailing pathogens causing a large number of deaths [34, 35]. Thus,* L. camara* leaf extracts can be more beneficial in treating most of these Gram negative disease causing pathogens. Methanol extract had the highest inhibition activity against all the tested microbes when compared to any other solvent extracts. The methanol leaf extract exhibited the highest activity against* E. coli* (24.1 ± 0.4 mm) followed by* K. pneumoniae* (18.1 ± 0.4 mm) at higher concentration (10 *μ*L/disc).* S. aureus* was also more vulnerable to methanol extract with 18.0 ± 0.4 mm of inhibition zone at 10 *μ*L/disc concentration. Among the tested bacterial strains,* E. coli* and* B. subtilis* showed increased zone of inhibition to all the solvent extracts and were the most susceptible strains. Ethyl extract was also most effective against* B. subtilis* while other bacterial strains were moderately inhibited. Likewise, acetone and chloroform extracts were also effective in inhibiting* E. coli* and* B. subtilis.* However, at lower concentrations, ethyl acetate extract showed no inhibition against* S. aureus*. In contrast, chloroform extract was less effective to all bacteria and at lower concentration, it failed to show antibacterial activity.* L. camara* finds its application in many parts of the world to cure various human ailments [7]. Previously, researchers have reported the varied antimicrobial potential of this plant and thus our reports support these findings [13, 14]. These results substantiate the findings of Naz and Bano [26] where methanol extract of* L. camara* leaves showed the highest antimicrobial activity. However, this is the first ever attempt which emphasizes the influence of different solvent extracts on human pathogenic bacterial strains. Our study clearly indicated the existence of considerable differences in the antibacterial activity among the various solvent extracts evaluated. This could be due to varied phytochemical constituents present in different solvent extracts.

**Figure 3:**
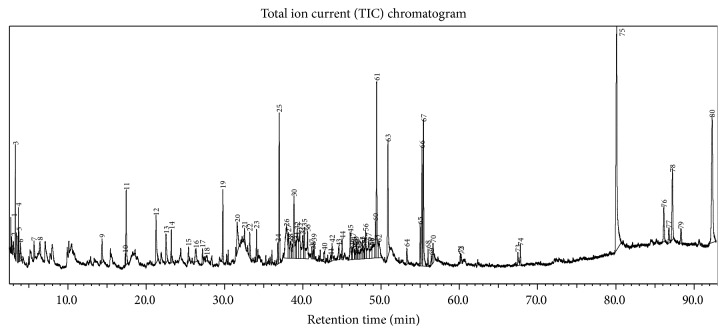
GC-MS chromatograph of methanolic leaf extract of* L. camara*.

Further, we used GC-MS analysis to identify the bioactive compounds occurring in the most competent solvent extract of* L. camara*. GC-MS profiling was performed only for methanolic leaf extract due to the fact that it contained many phytochemicals and exhibited superior biological activities. The distinctive chromatogram of the methanolic leaf extract of* L. camara* is shown in Figure 3. The analysis separated and identified a total of 32 known compounds belonging to different chemical classes (Table 5). The major compounds included hexadecanoic acid (5.197%), phytol (4.528%), caryophyllene oxide (4.605%), 9,12,15-octadecatrienoic acid, methyl ester, (Z,Z,Z)- (3.751%), 2,3-dihydro-2,5-dihydroxy-6-methyl-4H-pyran-4-one (2.954%), *α*-D-galactopyranoside, methyl (2.790%), coumaran (2.288%), germacrene-D (2.185%), bicyclo[5.2.0]nonane, 2-methylene-4,8,8-trimethyl (2.065%), spathulenol (1.888%), 1,2,3-propanetriol, 1-acetate (1.689%), propane-1,2,3-triol (1.1615), and 2,4(1H,3H)-pyrimidinedione, 5-methyl- (1.180%). Few other compounds identified in the extract are *α*-elemol, myristic acid, neophytadiene, furfuryl alcohol, propargyl alcohol, and acetic acid, fluoro-, ethyl ester. Many of these identified constituents are known to possess several pharmacological activities. Hexadecanoic acid, a major phytoconstituent of* L. camara* methanolic leaf extract, is known to possess strong antimicrobial activity [36]. The diterpene, phytol, is an important compound reported with antioxidant, cytotoxic, and antimicrobial properties [37]. Similarly a conjugated saponin, 2,3-dihydro-2,5-dihydroxy-6-methyl-4H-pyran-4-one, is reported to possess strong antioxidant, anticancer, and anti-inflammatory properties [38, 39]. Caryophyllene oxide, spathulenol, and germacrene-D are known to possess anticarcinogenic, anti-inflammatory, and antibacterial properties [2, 40]. As a biofumigant, coumaran is reported to act against insect pests found in stored food grains [41]. The compound 2,3-dihydro-2,5-dihydroxy-6-methyl-4H-pyran-4-one has been reported in plant extracts exhibiting antioxidant, antiproliferative, and anti-inflammatory properties [40]. More recently, anti-inflammatory and cytotoxicity activities of hexadecanoic acid, methyl ester have been reported by Othman et al. [42]. However, pharmacological activities of other compounds of* L. camara* methanolic leaf extract are yet to be determined. Therefore, we assume that the strong bioactivities exhibited by* L. camara* in this study are correlated to the occurrence of these bioactive compounds in the methanol solvent extract. However, further studies on the isolation, characterization, and biological evaluation of these identified compounds are necessary to confirm their potential benefits.

## 4. Conclusion

In conclusion, the present investigation clearly revealed that phytochemical composition of* L. camara* leaf extract varied with respect to different solvents. Total phenolic and flavonoid content significantly varied among the different solvent extracts. Methanol solvent extract of* L. camara* leaves contained more extractable metabolites compared to any other solvents. Moreover, all solvent extracts of* L. camara* showed considerable antioxidant and antimicrobial activity with varying differences due to differences in their phytochemical composition. Thus, our study suggests that methanol leaf extract of* L. camara* containing many bioactive compounds may possibly be utilized as a therapeutical source for developing beneficial drugs to manage various human diseases and disorders.

## Figures and Tables

**Table 1:** Qualitative screening of phytochemicals present in different solvent extracts of *L. camara* leaves.

Phytochemicals	Ethyl acetate	Methanol	Chloroform	Acetone
Alkaloids	−	+	−	+
Flavonoids	+	+	+	+
Saponins	+	+	−	+
Phenolics	+	+	+	+
Tannins	−	−	+	−
Anthraquinones	+	−	−	+
Cardiac glycosides	+	+	+	+
Terpenoids	−	+	+	−
Steroids	−	+	−	−

Note: + = present, − = absent.

**Table 2:** Dry weight and total yield of different solvent extracts of *L. camara* leaves.

Solvent extracts	Weight of the extract (*µ*g ± SD)	Yield (%)
Ethyl acetate	501.3 ± 3.5^b^	10.0
Methanol	721.3 ± 1.5^a^	14.4
Acetone	260.6 ± 4.0^c^	5.2
Chloroform	141.6 ± 2.5^d^	2.8

Note: each value is expressed as mean ± standard deviation (SD) (*n* = 3). Values in the column followed by a different letter superscript are significantly different (*p* < 0.05).

**Table 3:** Total phenolics and flavonoids content of different solvent extracts of *L. camara* leaves.

Solvent extracts	Total phenolic content	Flavonoid content
	(mg GAE/g) ± SD	(mg RE/g) ± SD
Ethyl acetate	75.6 ± 0.9^b^	16.7 ± 0.6^c^
Methanol	92.8 ± 1.7^a^	26.5 ± 0.5^a^
Acetone	62.9 ± 1.7^c^	20.6 ± 0.3^b^
Chloroform	33.7 ± 0.5^d^	21.2 ± 0.8^b^

Note: each value is expressed as mean ± standard deviation (SD) (*n* = 3). Values in the column followed by a different letter superscript are significantly different (*p* < 0.05) and values having the same letters are not statistically significant (*p* < 0.05). GAE: gallic acid equivalent, RE: rutin equivalent.

**Table 4:** Antibacterial activity of different solvent extracts of *L. camara *at different concentrations.

Solvent extracts (*µ*L/disc)	Zone of inhibition (mm)				
	*Escherichia coli*	*Klebsiella pneumoniae*	*Micrococcus luteus*	*Bacillus subtilis*	*Staphylococcus aureus*
Ethyl acetate					
2.5	08.2 ± 0.8	06.9 ± 0.5	06.1 ± 0.7	10.0 ± 0.5	—
5.0	13.0 ± 0.9	08.3 ± 0.5	07.1 ± 0.3	13.8 ± 0.5	06.1 ± 0.1
10.0	12.0 ± 0.5	10.5 ± 0.8	07.0 ± 0.5	13.9 ± 0.4	10.6 ± 0.4
Methanol					
2.5	14.6 ± 0.5	08.2 ± 0.3	08.1 ± 0.7	10.0 ± 0.3	14.1 ± 0.2
5.0	18.2 ± 0.2	14.5 ± 0.5	12.2 ± 0.3	14.0 ± 0.6	16.1 ± 0.6
10.0	24.1 ± 0.4	18.1 ± 0.4	18.0 ± 0.5	16.1 ± 0.2	18.0 ± 0.4
Acetone					
2.5	16.1 ± 0.4	06.1 ± 0.6	07.9 ± 0.2	14.2 ± 0.3	05.8 ± 0.6
5.0	24.0 ± 0.4	10.1 ± 0.6	12.3 ± 0.2	15.9 ± 0.4	10.2 ± 0.2
10.0	28.2 ± 0.6	16.2 ± 0.2	12.2 ± 0.2	16.3 ± 0.4	12.2 ± 0.4
Chloroform					
2.5	10.6 ± 0.6	—	—	10.5 ± 0.9	—
5.0	12.9 ± 0.6	09.2 ± 0.2	06.8 ± 0.7	12.1 ± 0.7	07.2 ± 0.2
10.0	14.8 ± 0.3	11.4 ± 0.8	08.2 ± 0.2	12.4 ± 0.4	07.9 ± 0.2

Note: the negative control discs were soaked with 50 *μ*L DMSO and the positive control discs with 50 *μ*L (50 *μ*g/mL) *chloramphenicol*. Each value represents the mean ± standard deviation (SD) of 3 replicates per treatment in 3 repeated experiments. “—” represents no activity.

**Table 5:** The major phytocompounds detected in the methanolic leaf extract of *L. camara* by GC-MS analysis.

S. number	Name of the compound	Peak number^*∗*^	Retention time (min)	Area (%)
1	2-Propanone, 1-hydroxy-	1	2.703	0.481
2	Propane-1,2,3-triol	3	3.275	1.161
3	Propargyl alcohol	4	3.653	0.877
4	Acetic acid, fluoro-, ethyl ester	5	3.719	0.737
5	Furfuryl alcohol	7	5.672	0.750
6	2,4(1H,3H)-Pyrimidinedione, 5-methyl-	9	14.366	1.180
7	2,3-Dihydro-2,5-dihydroxy-6-methyl-4H-pyran-4-one	11	17.443	2.954
8	Coumaran	12	21.251	2.288
9	1,2,3-Propanetriol, 1-acetate	13	22.529	1.689
10	Cyclohexasiloxane, dodecamethyl-	14	23.200	0.978
11	4-Vinylguaiacol	15	25.393	0.809
12	Bicyclo[5.2.0]nonane, 2-methylene-4,8,8-trimethyl	19	29.798	2.065
13	Germacrene-D	21	32.547	2.185
14	Longifolene	22	33.22	0.889
15	4-Epi-cubedol	23	34.084	0.817
16	Caryophyllene oxide	25	36.995	4.605
17	*α*-D-Galactopyranoside, methyl	26	37.909	2.790
18	Humulene epoxide II	27	38.173	0.696
19	Spathulenol	30	38.886	1.888
20	*α*-Elemol	34	39.997	0.507
21	Myristic acid	42	43.751	0.652
22	Neophytadiene	45	46.059	0.820
23	3-Eicosyne	53	47.594	0.211
24	9-Octadecenoic acid (Z)-, methyl ester	60	49.296	0.093
25	Hexadecanoic acid, methyl ester	61	49.438	0.696
26	Hexadecanoic acid	63	50.888	5.197
27	9,12-Octadecadienoic acid (Z,Z)-, methyl ester	65	55.007	1.037
28	9,12,15-Octadecatrienoic acid, methyl ester, (Z,Z,Z)-	66	55.225	3.751
29	Phytol	67	55.426	4.528
30	Octadecanoic acid, methyl ester	68	56.428	0.330
31	9,12,15-Octadecatrienoic acid, (Z,Z,Z)-	70	56.640	1.019
32	Phthalic acid, di(2-propylpentyl) ester	74	67.804	0.688

^*∗*^Peak number is represented in Figure 3.
